# Habitual night sleep duration is associated with general obesity and visceral obesity among Chinese women, independent of sleep quality

**DOI:** 10.3389/fpubh.2023.1053421

**Published:** 2023-01-23

**Authors:** Jiangshan He, Yaqi Fan, Li Zhang, Chunjun Li, Fenghua Guo, Jiahui Zhu, Pei Guo, Binbin Zhang, Mianzhi Zhang, Minying Zhang

**Affiliations:** ^1^School of Medicine, Nankai University, Tianjin, China; ^2^Health Management Centre, Tianjin First Central Hospital, Tianjin, China; ^3^Tianjin Union Medical Center, Tianjin, China; ^4^Dongfang Hospital, Beijing University of Chinese Medicine, Beijing, China; ^5^Department of Nephrology, Tianjin Academy of Traditional Medicine Affiliated Hospital, Tianjin, China

**Keywords:** sleep duration, body mass index, percentage of body fat, visceral fat area, women

## Abstract

**Purpose:**

Research on the relationship between sleep duration and obesity defined using multiple anthropometric and bioelectrical indices in women remains scarce. We aimed to explore the association between sleep duration and body mass index (BMI), waist-hip ratio (WHR), body fat percentage (PBF) and visceral fat area (VFA) among females.

**Methods:**

We recruited women for medical examination using multistage cluster sampling. Sleep was assessed using Pittsburgh Sleep Quality Index (PSQI) and sleep duration was categorized into short (<7 h), optimal (7 <9 h) and long sleep (≥ 9 h). Weight and height were measured using a calibrated stadiometer. Waist circumference was manually measured. PBF, and VFA were estimated by bioelectrical impedance analysis. Data on sociodemographic characteristics and lifestyle factors were also collected and included in the logistic regression models to explore the independent association between sleep duration and obesity defined by different indices.

**Results:**

A total of 7,763 women with a mean age of 42.6 ± 13.5 years were included. The percentage of women reporting short and long sleep was 10.3 and 13.4% respectively. The mean BMI, WHR, PBF and VFA were 23.07 ± 3.30 kg/m^2^, 0.78 ± 0.06, 32.23 ± 6.08% and 91.64 ± 35.97cm^2^, respectively. Short sleep was independently associated with 35% (95% CI: 1.05–1.75) increased odds of general obesity (BMI ≥ 28 kg/cm^2^), and long sleep was associated with 18% (95% CI: 1.01–1.37) increased odds of visceral obesity (VFA > 100 cm^2^). No association was observed between sleep deprivation or excessive sleep and high WHR or high PBF.

**Conclusion:**

In women, short sleep was associated with an increased odds of general obesity, whereas long sleep was associated with an increased odds of visceral obesity. Longitudinal observations are needed to confirm this cross-sectional relationship.

## 1. Introduction

The increasing prevalence of obesity has posed significant challenges for public health around the world (1). In parallel, chronic sleep restriction and poor sleep quality raises public health concerns related to both safety and health globally. In China, approximately a quarter of adults sleep <7 h per night (2).

Optimal sleep duration plays an important role in maintaining metabolic homeostasis. Studies suggested that chronic sleep deprivation or excessive sleep was associated with increased risk of common chronic diseases (e.g., hypertension, type 2 diabetes and metabolic syndrome) and all-cause mortality (3–6). The evidence for chronic sleep deprivation as a risk factor for obesity is strong (7, 8). Possible biological mechanisms have been indicated that short sleep duration has adverse impact on metabolism, resulting in damaged metabolic homeostasis and obesity (9, 10).

Though Body mass index (BMI) is the most widely used index to define obesity, it is inefficient in predicting the adverse impact on health of excess adiposity accumulation for it could not distinguish between fat and muscle (7). Research has suggested that not only the mass but also the area of fat accumulation was associated with the unfavorable impact on health (11). Percentage of body fat (PBF), waist-to-hip ratio (WHR) and visceral fat area (VFA) outperform BMI by providing additional information on the body fat content, central obesity, and visceral obesity, respectively (12–14). Therefore, the use of multiple anthropometrics to define obesity has both clinical and practical implications.

Though the evidence for the association of chronic sleep deprivation with obesity is strong, (15) the relationship between sleep duration and obesity among females has not been thoroughly explored and the current evidence is inconsistent (16, 17). In China, the prevalence of obesity for women reached 13.37% with an upward trend (18). Due to physiological cycle changes such as menstruation, pregnancy and menopause, women's sleep was affected by different factors from childhood to menopause. A meta-analysis has shown that women have a 41% higher risk of developing insomnia than men (19). Research indicated that sex modified the association between sleep duration and obesity (20, 21). The circadian system plays a role in the regulation of metabolic homeostasis, circadian desynchrony may be involved in the development of obesity and metabolic disorders. Research has indicated that sex modifies the association between the clock circadian regulator (*CLOCK*) gene, a core component of the circadian system, and BMI (21).

As numerous studies exploring the relationship between sleep duration and obesity focused on BMI and the results stratified by gender are inconsistent (16, 17) an improved understanding of the association between sleep duration and obesity defined by various indices among women may improve outcomes. The current study therefore aimed to explore the relationship between sleep duration and obesity defined by BMI, PBF, WHR and VFA in non-pregnant and non-breastfeeding women. We used part of the baseline data of the Medical Examination Cohort in Beijing-Tianjin-Hebei Region, China, which was designed to provide reliable data for the health conditions and associated factors among adults.

## 2. Materials and methods

### 2.1. Study design and population

This study analyzed part of the baseline data of the Beijing-Tianjin-Hebei Medical Examination Cohort (BTH-MEC), a national key R&D program of China. The study design and population have been reported elsewhere (22). The participants were recruited by multistage stratified cluster sampling among adults for medical examinations from August 2018 to December 2020. Individuals aged 18 years or older who voluntarily participated in the survey were included in the research. Individuals were excluded if they (1) were with cognitive impairment, hearing impairment, articulate problems, or severe mental illness and could not complete the survey; (2) were with heart pacemakers implanted; and (3) could not stand independently. We analyzed the data of female participants in Tianjin. With a response rate of 86.5%, 7,879 women were interviewed and underwent medical examinations and 7,763 were included in the analysis after excluding those who were pregnant (*n* = 3), lactating (*n* = 1), taking weight-loss drugs (*n* = 3) in the prior 3 months or missing one or more variables of interest (*n* = 109). The research protocol was reviewed and approved by ethical committees from Nankai University (NKUIRB2016063), and written informed consent was got from each participant. The research procedures were carried out strictly following the Declaration of Helsinki. All methods were carried out in accordance with relevant guidelines and regulations.

### 2.2. Outcome variables

Height (0.1 cm precision) and weight (0.1 kg precision) was measured without shoes or heavy clothes using a calibrated stadiometer (GL-310, Seoul, Korea). Waist circumference (WC) and hip circumference was manually measured by medical professionals. PBF, and VFA were estimated by bioelectrical impedance analysis (BIA) using the multifrequency impedance plethysmograph body composition analyzer (Inbody-770, Biospace, Seoul, Korea). The age, sex and height of participants were input into the system prior to the assessment. The participant stood barefoot on the foot electrode of the instrument in a fully vertical position with light clothing, shared the weight evenly on both legs, held the hand electrode with both hands, and was prohibited from speaking during measurement. Measurement was completed after the reading was stable, as reported elsewhere (23).

According to Chinese guidelines, general obesity was defined as BMI ≥ 28 kg/m^2^ for Chinese women (24, 25). The WHO report suggests high WHR was defined as WHR ≥ 0.80 for Asian females (26). Visceral obesity was defined as VFA ≥ 100 cm^2^ (27). Due to the lack of criteria on high PBF obesity, we defined high PBF as > 35% in women based on previous literature (28).

### 2.3. Sleep duration

We used the Pittsburgh Sleep Quality Index (PSQI) (29) to assess the participants' habitual sleep duration and sleep quality. The Chinese version of the PSQI has been shown to have good reliability and validity among Chinese adults (30–32). Higher global PSQI score indicates poor sleep quality, whereas a cutoff of ≤ 7 indicating good sleep quality was suggested to have high diagnostic sensitivity and specificity (98.3 and 90.2%, respectively) in the Chinese population (30). According to the National Sleep Foundation, the optimal sleep duration for adults is 7–9 h per night (33). However, a few participants reported sleeping > 9 h in this study, so we categorized sleep duration into <7 h, 7–9 h (including 7 h but not including 9 h) and ≥ 9 h. Subjective sleep quality (very good, fairly good, fairly bad and very bad) and usage of sleep medicine (not during the past month, less than once a week, once or twice a week, and three or more times a week) was also collected using PSQI.

### 2.4. Covariates

Trained investigators conducted a face-to-face questionnaire to collect data on health related covariates which were also included in the multivariate logistic regression models including demographic characteristics (age, sex, occupation, education level, and marital status), lifestyle factors (smoking status), alcohol drinking, physical exercise, sedentary (being sitting or reclining) duration, personal history of chronic diseases (hypertension, diabetes, coronary heart disease, dyslipidemia, stroke, chronic kidney disease, chronic obstructive pulmonary disease) and cancer, medication taken in the prior 3 months (such as lowering uric acid agents, anti-arrhythmia agents, hormone agents and sleeping agents).

### 2.5. Statistical analyses

The normally distributed continuous variables were expressed as mean ± standard deviation (SD). Categorical data were described as rate and proportion. Differences in characteristics of participants across sleep duration categories were compared with Chi-square test and Kruskal–Wallis test. We conducted one-way ANOVA to examine the differences in BMI, WHR, PBF and VFA across sleep duration categories, and Tukey's test was used for pairwise multiple comparison. By including obesity indices as binary outcomes [i.e., general obesity (BMI ≥ 28 kg/m^2^) or not, elevated WHR (WHR ≥ 80) or not, high PBF (PBF > 35%) or not, and visceral obesity (VFA ≥ 100 cm^2^) or not], we conducted multivariate logistic regression to explore the associations between sleep duration, sleep quality with obesity defined by different indices. As proposed by previous literature (5, 34, 35) covariates that may act as confounder such as sociodemographic characteristics (age, occupation, marriage status, education level), lifestyles (smoking, alcohol drinking, exercise and sedentary duration), personal history of chronic diseases, regular medication taken in the prior 3 months, and subjective sleep quality and usage of hypnotic were included in the regression models to address the potential confounding. Odds ratios (ORs) and 95% confidence intervals (CIs) were calculated to measure the risk magnitude of sleep duration, sleep quality for each type of obesity. We constructed Model 1 to examine the associations between sleep duration and obesity defined by different indices, Model 2 to examine the associations between sleep quality and obesity defined by different indices. Model 3 examined the associations of sleep duration and sleep quality with obesity defined by different indices. All analyses were performed using SPSS (version 25.0) software. All tests were 2-sided, with statistical significance set at *p* < 0.05.

## 3. Results

A total of 7,763 non-pregnant and non-breastfeeding women aged 18–89 years with a mean of 42.6 ± 13.5 years were included in the present study. Characteristics of the participants according to the categories of sleep duration were presented in (Table 1). In total, 10.3 and 13.4% reported sleeping <7 h and ≥ 9 h per night respectively. Age, occupation, smoking status, physical exercise, daily sedentary duration, subjective sleep quality and taking hypnotic were all correlated with sleep duration (all *p* < 0.05), whereas marriage status, education level, alcohol drinking, history of chronic diseases, having regular medication in the prior 3 months were not found to be associated with sleep duration (all *p* > 0.05).

**Table 1:** Characteristics of study participants by sleep duration groups (*n* = 7763).

**Variables**	**Sleep duration**			**P-value**

	<**7 h (*****n** =* **796)**	**7–9 h (*****n** =* **5925)**	≥**9 h (*****n** =* **1042)**	
**Age (years)**				<0.001
18–44	438 (9.3%)	3597 (76.0%)	695 (14.7%)	
45–64	283 (11.8%)	1840 (77.0%)	268 (11.2%)	
65–89	75 (11.7%)	488 (76.0%)	79 (12.3%)	
**Occupation**				0.009
Professionals/technicians	500 (11.0%)	3443 (76.0%)	588 (13.0%)	
Clerical support workers	182 (9.7%)	1457 (77.3%)	244 (13.0%)	
Others	114 (8.5%)	1025 (76.0%)	210 (15.5%)	
**Marriage status**				0.114
Unmarried	108 (8.6%)	970 (77.7%)	171 (13.7%)	
In a current marriage	660 (10.4%)	4813 (76.2%)	846 (13.4%)	
Divorced/widowed	28 (14.4%)	141 (72.7%)	25 (12.9%)	
**Education level**				0.465
High school/below	71 (11.2%)	480 (75.9%)	81 (12.8%)	
College/university	477 (10.3%)	3494 (75.8%)	640 (13.9%)	
Postgraduate/above	246 (9.8%)	1951 (77.5%)	321 (12.7%)	
**Smoking**				<0.001
Never	729 (9.9%)	5627 (76.5%)	1003 (13.6%)	
Former	12 (12.2%)	76 (77.6%)	10 (10.2%)	
Current	55 (18.0%)	221 (72.5%)	29 (9.5%)	
**Alcohol drinking**				0.165
Never	712 (10.0%)	5426 (76.5%)	957 (13.5%)	
Former	6 (17.1%)	27 (77.1%)	2 (5.7%)	
Current	78 (12.3%)	472 (74.6%)	83 (13.1%)	
**Exercise**				<0.001
Never	349 (12.8%)	2075 (76.0%)	307 (11.2%)	
Occasional	274 (8.4%)	2488 (75.6%)	528 (16.0%)	
Regular	172 (9.9%)	1362 (78.2%)	207 (11.9%)	
**Sedentary duration**				<0.001
≤ 2 h	32 (14.3%)	151 (67.4%)	41 (18.3%)	
2.1–4 h	98 (10.0%)	713 (73.1%)	165 (16.9%)	
4.1–6 h	225 (9.3%)	1879 (77.7%)	313 (12.9%)	
> 6 h	441 (10.6%)	3182 (76.7%)	523 (12.6%)	
**Sleep quality**				<0.001
Good	698 (9.31)	5768 (76.97)	1028 (13.72)	
Poor	98 (36.43)	157 (58.36)	14 (5.21)	
**Subjective sleep quality**				<0.001
Good and excellent	609 (9.3%)	5019 (76.8%)	911 (13.9%)	
Bad and awful	187 (15.3%)	904 (74.0%)	130 (10.6%)	
**Taking hypnotic**				<0.001
Never	718 (9.7%)	5666 (76.7%)	1003 (13.6%)	
Once a week	29 (23.0%)	85 (67.5%)	12 (9.5%)	
Twice a week	18 (20.5%)	63 (71.6%)	7 (8.0%)	
≥ 3 times a week	31 (19.5%)	108 (67.9%)	20 (12.6%)	
**Chronic diseases**				0.126
Yes	135 (11.6%)	883 (76.0%)	144 (12.4%)	
No	657 (10.0%)	5032 (76.4%)	896 (13.6%)	
Unknowing	4 (25.0%)	10 (62.5%)	2 (12.5%)	
**Cancer**				0.111
Yes	15 (13.9%)	87 (80.5%)	6 (5.6%)	
No	779 (10.2%)	5828 (76.3%)	1032 (13.5%)	
Unknowing	2 (18.2%)	5 (45.4%)	4 (36.4%)	
**Regular medication in the prior three months**				0.389
Yes	54 (11.4%)	363 (76.9%)	55 (11.7%)	
No	742 (10.3%)	5562 (76.3%)	987 (13.5%)	
BMI ≥ 28 kg/m^2^	82 (10.3%)	447 (7.54%)	78 (7.49%)	0.023
WHR ≥ 0.80	384 (48.2%)	2873 (48.5%)	510 (48.9%)	0.950
PBF > 35%	255 (32.0%)	1873 (31.6%)	333 (31.9%)	0.887
VFA ≥ 100 cm^2^	305 (38.3%)	2112 (35.6%)	393 (37.7%)	0.130

Data were presented as n (%). BMI: body mass index; WHR: waist-hip ratio; PBF: body fat percentage; VFA: visceral fat area. Sleep quality was assessed according to global PSQI score. Higher score indicates poor sleep quality, whereas a cutoff of ≤ 7 indicating good sleep quality.

The prevalence of general obesity (BMI ≥ 28 kg/m^2^), high WHR and PBF, and visceral obesity were 7.82, 48.53, 31.70, and 36.20%, respectively. The prevalence of high WHR, PBF, and VFA were considerably higher than that of general obesity. As shown in Table 1, the highest prevalence of general obesity, high PBF and visceral obesity was observed in short sleep group, while the highest prevalence of high WHR was observed in long sleep group. Only women reported sleeping <7 h had a higher prevalence of general obesity than that of optimal and long sleep (*p* < 0.05). The prevalence of high WHR, PBF and VFA were not statistically different across sleep durations (all *p* > 0.05).

The mean BMI, WHR, PBF and VFA among the total population and women sleeping <7 h, 7–9 h and ≥ 9 h was presented in (Table 2). Women reporting short sleep had higher BMI than that of women reporting 7–9 h of sleep (*p* = 0.010) and > 9 h of sleep (*p* = 0.028), whereas WHR, PBF and VFA were not significantly different across sleep duration categories.

**Table 2:** Obesity measurements of study participants by sleep duration groups (*n* = 7763).

**Measurements**	**Total population**	**Sleep duration**			**P-value**

		<**7 h (*****n** =* **796)**	**7–9 h (*****n** =* **5925)**	≥**9 h (*****n** =* **1042)**	
BMI (kg/m^2^)	23.07 ± 3.30	23.36 ± 3.46^a^	23.04 ± 3.28^b^	23.02 ± 3.30^b^	0.03
WHR	0.78 ± 0.06	0.78 ± 0.04^a^	0.78 ± 0.06^a^	0.78 ± 0.07^a^	0.46
PBF (%)	32.23 ± 6.08	32.22 ± 6.40^a^	32.21 ± 6.05^a^	32.33 ± 6.05^a^	0.83
VFA (cm^2^)	91.64 ± 35.97	92.93 ± 37.70^a^	91.36 ± 35.64^a^	92.26 ± 36.50^a^	0.44

Data were presented as mean ± (SD). BMI, body mass index; WHR, waist-hip ratio; PBF, body fat percentage; VFA, visceral fat area. ^a, b^Designated for *post hoc* analysis. The same letter indicates that the difference between the two groups is not statistically significant, while different letters indicate that the difference is statistically significant.

Table 3 showed the ORs and 95% CIs of sleep duration for obesity defined by BMI, WHR, PBF and VFA. After completely adjusting for sleep quality and all selected covariates (age, occupation, marriage status, education level, smoking, alcohol drinking, exercise, sedentary duration, personal history of chronic diseases, regular medication taken in the prior 3 months, subjective sleep quality, usage of hypnotic and sleep quality), sleep <7 h was associated with 35% elevated odds of general obesity (95% CI: 1.04–1.74), whereas sleep ≥ 9 h was associated with 19% elevated odds of visceral obesity (95% CI: 1.03–1.38). In addition, short sleep demonstrated a marginally significant association with high WHR (OR = 0.83, 95% CI: 0.69–1.00). No statistically significant correlations were observed between short sleep and high PBF or visceral obesity, long sleep and general obesity, high PBF or high WHR. We did not observe any significant association between sleep quality and obesity defined by any of the studied adiposity indicators.

**Table 3:** The association between sleep duration and obesity defined by different indices.

**Obesity**	**Model 1 OR (95% CI; P-Value)**	**Model 2** **OR (95% CI; P-Value)**	**Model 3 OR (95% CI; P-Value)**
**General obesity**			
Sleep duration			
<7 h	1.35 (1.05–1.75; 0.02)		1.35 (1.04–1.74; 0.02)
7–9 h	Reference		Reference
≥ 9 h	0.99 (0.76–1.27; 0.91)		0.99 (0.76–1.27; 0.91)
Sleep quality			
Good		0.86 (0.51–1.46; 0.75)	0.94 (0.55–1.60; 0.82)
Poor		Reference	Reference
**High WHR**			
Sleep duration			
<7 h	0.84 (0.70–1.01; 0.06)		0.83 (0.69–1.00; 0.05)
7–9 h	Reference		Reference
≥ 9 h	1.16 (0.99–1.36; 0.08)		1.16 (0.99–1.36; 0.07)
Sleep quality			
Good		0.88 (0.55–1.41; 0.59)	0.80 (0.50–1.30; 0.37)
Poor		Reference	Reference
**High PBF**			
Sleep duration			
<7 h	0.97 (0.82–1.14; 0.68)		0.96 (0.81–1.14; 0.67)
7–9 h	Reference		Reference
≥ 9 h	1.06 (0.92–1.24; 0.41)		1.05 (0.91–1.22; 0.41)
Sleep quality			
Good		0.99 (0.70–1.40; 0.94)	0.97 (0.69–1.38; 0.86)
Poor		Reference	Reference
**Visceral obesity**			
Sleep duration			
<7 h	1.08 (0.91–1.28; 0.37)		1.08 (0.91–1.27; 0.40)
7–9 h	Reference		Reference
≥ 9 h	1.19 (1.03–1.38; 0.02)		1.19 (1.03–1.38; 0.02)
Sleep quality			
Good		0.95 (0.66–1.37; 0.79)	0.95 (0.66–1.37; 0.79)
Poor		Reference	Reference

WHR, waist-hip ratio; PBF, body fat percentage. General obesity was defined as BMI ≥ 28 kg/m^2^. Model 1: Examine the association between sleep duration and obesity defined by different indices adjusted for age, occupation, marriage status, education level, smoking, alcohol drinking, exercise, sedentary duration, personal history of chronic diseases, regular medication taken in the prior three months, subjective sleep quality and usage of hypnotic. Model 2: Examine the association between sleep quality and obesity defined by different indices adjusted for the same variables as Model 1. Model 3: Same as Model 1 but also adjusted for sleep quality.

## 4. Discussion

In the present study, we found short sleep was associated with general obesity, and excessive sleep was associated with visceral obesity among females. Women who slept <7 h had 35% increased odds of general obesity, while women who slept ≥ 9 h had 18% increased odds of visceral obesity compared with those sleeping 7–9 h per night. But no statistically significant association was observed between sleep duration and PBF or WHR.

An association between sleep and obesity has been suggested in a substantial body of research, but many previous studies used BMI or other conventional anthropometric indices [e.g., waist circumference (WC), WHR and waist-to-height ration (WHtR)] as adiposity measures (36, 37). It has been suggested that not only the mass but also specific anatomical locations of adipose depots are associated with the adverse impact on health (38). Visceral fat is highly metabolically active and is constantly releasing free fatty acids (FFA) into the portal circulation. As such, visceral fat content contributes to various features of the metabolic syndrome, such as hyperinsulinemia, systemic inflammation, dyslipidemia, and atherosclerosis (39, 40). In addition, visceral fat showed significant positive correlations with cardiovascular risk factor markers (41). In the current study, the prevalence of higher PBF, WHR, and visceral obesity were much higher than that of BMI, suggesting that BMI is not sufficient to identify the excess fat accumulation and the related health risk, a better understanding of the association of sleep duration with obesity defined by various indices may improve outcomes.

At present, several studies have concluded that lack of sleep is related to general obesity in adults, but the results have been inconsistent among women. Being consistent with our findings, studies conducted in Korea and America reported that short sleep was associated with the 18 and 22% increased odds of general obesity in women, respectively (16, 42). But, the study conducted among employees at a Japanese electric power company didn't observe a statistical association between short sleep and general obesity in women (43). Study results on the relationship between sleep duration and PBF had also been mixed. Women who slept for <6 h were found to have an increased risk of high PBF by a study from Urumqi, China (44). However, this study used 6–8 h of sleep as the reference, and high PBF was defined as a PBF higher than 30%, which were different from our study and may account for the inconsistent findings. Being consistent with our research, several studies have not found any relationship between sleep duration and PBF or WHR in women, including research using objectively measured sleep duration (45, 46). In the current study, no statistically significant association was observed between short sleep and VFA, several studies found similar results, whereas others reported positive results (23, 47). Research from China found the adjusted odds ratio for visceral obesity was 1.22 (95% CI: 1.02–1.45) in women sleeping <7 h compared with those sleeping 7–9 h, after adjusting for all confounding factors (23). Inconsistent with the current findings, our previous study linked short sleep with visceral obesity (OR = 1.22 95% CI: 1.02–1.45) (23). The different findings may be partially explained by the fact that the current study included more young women (60.9%) who had longer sleep and less visceral obesity than the previous. Age, independent of other risk factors, plays a fundamental role in both visceral obesity and sleep among females, especially in postmenopausal women. The higher proportion of young long sleepers and the lower proportion of visceral obesity in our study population may have made the association between long sleep and visceral obesity less statistically significant. Though the OR of chronic sleep deprivation for visceral obesity is not statistically significant in the present study, it is greater than the reference, suggesting that short sleep might be a potential risk factor for visceral obesity.

Though research reported a U-shaped relationship between sleep duration and BMI among adults, no association was found between long sleep, BMI, PBF and WHR among women in the present study (9). However, long sleep was found with 18% increased odds of visceral obesity compared with optimal sleep among women. A Swedish study found that long sleep could increase the risk of general obesity in women but decrease the risk of general obesity in men (21). Another study showed that long sleep females (≥ 9 h) demonstrated a higher prevalence of higher PBF (OR = 1.43, 95% CI: 1.04–1.96) compared with those who slept 7–8h (48). However, few studies have examined the relationship between sleep duration and WHR and VFA (46). The current study demonstrated a significant association between long sleep and elevated VFA, and a marginally significant association between long sleep and high WHR. Though research in Japan suggested that sleep duration was not related to visceral fat area and research in Korea demonstrated that the adjusted mean VFA and hepatic fat were highest in the shortest sleep duration group (<5 h) and decreased linearly with increasing sleep duration (49, 50). We found long sleep is associated with elevated odds of visceral obesity, which is similar to our previous study where the OR of long sleep for visceral obesity is marginally significant (OR = 1.10, 95% CI: 0.92–1.32) (23). We speculated the different populations, cutoff values of short or long sleep might contribute to the inconsistent findings. More longitudinal data is needed to further validate this finding.

Although, the pathophysiology of sleep duration and obesity has not yet been fully explored, several mechanisms have been proposed to explain the relationship between short sleep duration and obesity. First, a number of studies have shown that short sleep duration may lead to the decrease of leptin level and the increase of ghrelin level, which leads to the increase of hunger and appetite (9, 51). Chronic sleep restriction may contribute to the long-term hormone alteration (9). Second, chronic sleep deprivation leads to a decrease in melatonin levels, which affects brown adipose tissue replenishment and metabolism, therefore reduces energy expenditure and involves in the development of obesity (52). Third, shorter sleep duration is associated with obesity-related behaviors, changing the quantity, composition and distribution of the daily diet, consuming energy from snacks rather than regular meals, eating fewer vegetables and fruits, and feeling fatigue leading to reduced physical activity (53). At present, there was still few evidence on the potential mechanism of the association between long sleep and visceral obesity. Further studies are needed to investigate the underlying mechanism of this relationship and its implication.

We did not observe any significant relationship between sleep quality and obesity defined by the studied indices. Similar results were found in a study among Turkish women (54). However, studies in Chinese reproductive-aged women, middle-aged Italian adults, and German adults have linked sleep quality with WC, BMI and WC, and BMI and body fat mass, respectively (55–57). Longitudinal observations are needed to further explore the association between sleep quality and obesity defined by various adiposity indices.

Cautions should be taken when comparing our findings with other research for different criteria were used for short sleep. Many of the mentioned categorized short sleep as <6 h and even more extreme sleep restriction (≤ 5 h) (16, 43, 45–47). Because very few participants in our study reported <6 h of sleep, we then used 7–9 h as the optimal sleep duration and short sleep was categorized as <7 h. Despite the wide range of short sleep duration, positive results were observed regarding the unfavorable effect of <7 h sleep in the current study. The criteria we use for categorizing sleep duration was supported by the Consensus Statement released by the American Academy of Sleep Medicine and Sleep Research Society. In general, there was consensus that 6 h of sleep or less was associated with unfavorable health outcome, while 7–9 h of sleep were appropriate to support optimal health in adults. Consensus could not be reached on the appropriateness of 6–7 h of sleep, but the median vote suggested this duration was in the inappropriate range and the minimum threshold was set at 7 h as the lowest sleep duration appropriate to support optimal health in adults (58). Nevertheless, a narrower range for the sleep duration category was recommended for use in future studies that include sufficient numbers of people with <6 h of sleep to find a more precise link between sleep duration and obesity.

There were several strengths in the current study. First, the study was the first to investigate the relationship between sleep duration and VFA among Chinese females as far as we know. Second, we adjusted for subjective sleep quality and taking hypnotics as covariates. Prior study had shown that self-reported short sleep duration might be a sign of subjective poor sleep quality (59) and taking hypnotics might affect sleep duration, therefore, including subjective sleep quality and taking hypnotics as a covariate could help to evaluate the relationship between sleep duration and adiposity indices more accurately. Furthermore, short and long sleep was reported to be associated with chronic disease, which may have mixed effects on the relationship between sleep duration and obesity. In the current study, chronic disease status of the participants was also collected and included as covariate in the analysis to improve the accuracy and reliability of the study results.

However, our study also had several limitations. First, the cross-sectional nature of this study prevented a causal interpretation for sleep duration and adiposity indices. As obesity plays an essential role in the pathophysiology of sleep disorders and evidence on the bilateral and mutual interactions linking sleep and metabolic disorders is advanced, the observed correlations between sleep duration and obesity might be supportive for a reverse causation (60–62). Second, sleep duration in this study was self-reported rather than objective measured. However, measurement of objective sleep duration is not applicable for large sample studies for it needs participants to wear wrist activity monitors. Previous research had shown the consistency of sleep parameters derived from subjective questionnaires and those derived from polysomnography (63). Third, visceral fat area was measured by BIA rather than computed tomography and magnetic resonance imaging, However, CT and MRI need expensive and specialized equipment, and require participants to be exposed to radiation, which are not applicable for screening large size of general population. Finally, the participants were recruited at medical examination centers based on voluntary participation. The young age of the study population and the high proportion of desk jobs limits the generalizability of the current findings.

## 5. Conclusion

In conclusion, our results add to the current evidence to suggest that short sleep was associated with elevated odds of general obesity, whereas long sleep was associated with elevated odds of visceral obesity in women. Most research has suggested an association between sleep duration and obesity as defined by BMI, however, our results suggest that the association also involves obesity as defined by measures other than BMI, demonstrating the importance of using multiple adiposity indices to identify obesity associated with sleep duration. Prospective studies with various measures of adiposity are needed to confirm the observed cross-sectional correlations between sleep duration and obesity.

## Data availability statement

The raw data supporting the conclusions of this article will be made available by the authors, without undue reservation.

## Ethics statement

The studies involving human participants were reviewed and approved by the Tianjin First Central Hospital (No. 2017N052KY) and Tianjin Union Medical Center (No. 2018C02). The patients/participants provided their written informed consent to participate in this study.

## Author contributions

MinZ has designed the study, supervised and oversaw the study implementation, wrote the manuscript, the guarantors of this work, had full access to all of the data in the study, and take responsibility for the integrity of the data and the accuracy of the data analysis. JH conducted the investigation and participated in writing and revising the manuscript. YF conducted the analysis of the data. LZ participated in writing the manuscript. LZ, CL, and MiaZ have organized and managed the investigation. JH, YF, JZ, PG, BZ, CL, JZ, and MiaZ have participated in the investigation and the management of the data. All authors contributed substantially to the study, read, and approved the final manuscript.
